# Classification of abnormal location in medium voltage switchgears using hybrid gravitational search algorithm-artificial intelligence

**DOI:** 10.1371/journal.pone.0253967

**Published:** 2021-07-01

**Authors:** Hazlee Azil Illias, Ming Ming Lim, Ab Halim Abu Bakar, Hazlie Mokhlis, Sanuri Ishak, Mohd Dzaki Mohd Amir

**Affiliations:** 1 Department of Electrical Engineering, Faculty of Engineering, Universiti Malaya, Kuala Lumpur, Malaysia; 2 Centre of Advanced Manufacturing & Material Processing (AMMP Centre), Faculty of Engineering, Universiti Malaya, Kuala Lumpur, Malaysia; 3 UM Power Energy Dedicated Advanced Centre (UMPEDAC), Level 4, Wisma R&D UM, Universiti Malaya, Kuala Lumpur, Malaysia; 4 TNB Research Sdn. Bhd., No. 1, Kawasan Institusi Penyelidikan, Kajang, Selangor, Malaysia; Universiti Sains Malaysia, MALAYSIA

## Abstract

In power system networks, automatic fault diagnosis techniques of switchgears with high accuracy and less time consuming are important. In this work, classification of abnormal location in switchgears is proposed using hybrid gravitational search algorithm (GSA)-artificial intelligence (AI) techniques. The measurement data were obtained from ultrasound, transient earth voltage, temperature and sound sensors. The AI classifiers used include artificial neural network (ANN) and support vector machine (SVM). The performance of both classifiers was optimized by an optimization technique, GSA. The advantages of GSA classification on AI in classifying the abnormal location in switchgears are easy implementation, fast convergence and low computational cost. For performance comparison, several well-known metaheuristic techniques were also applied on the AI classifiers. From the comparison between ANN and SVM without optimization by GSA, SVM yields 2% higher accuracy than ANN. However, ANN yields slightly higher accuracy than SVM after combining with GSA, which is in the range of 97%-99% compared to 95%-97% for SVM. On the other hand, GSA-SVM converges faster than GSA-ANN. Overall, it was found that combination of both AI classifiers with GSA yields better results than several well-known metaheuristic techniques.

## 1. Introduction

Switchgears are used to protect power system networks from equipment failures in the event of faults. Switchgear failures are rare but when they occur, it can cause serious injury and major damage. Thus, diagnosis and condition monitoring of switchgears are important in assessing the location of abnormality within the equipment [1, 2]. Commonly methods used for monitoring are on-line and off-line techniques. On-line monitoring techniques do not require a switchgear to be disconnected from the power supply during the equipment monitoring. With the emergence of sensor development and signal processing techniques nowadays, on-line fault diagnosis and condition monitoring of switchgears are commonly used and the technology associated with it are widely researched.

Several techniques are available for condition monitoring of switchgears, which include visual inspection, infrared thermography, transient earth voltage and ultrasound level [3, 4]. New techniques based on passive, surface acoustic waves and wireless sensors have effectively reduced the installation cost and enhanced monitoring by allowing measurements at unreachable locations [3, 5–7]. These technologies can help in diagnosis of switchgears efficiently, accurately and intelligently, which in turn enhances the intelligence of power grid, especially in smart grid. Smart grid consists of many high performance electronic devices and advanced monitoring technologies, which require reliable information and communication technologies.

Sulphur hexafluoride (SF_6_) has been widely applied as insulation of gas insulated switchgears (GIS) in power systems [8, 9]. The usage of SF_6_ can reduce the size and cost of GIS and improve the operation and maintenance of GIS. However, any abnormal condition on the insulation in GIS can cause partial discharge (PD), which could lead to insulation breakdown [2, 6, 10]. Hence, detection of PD within switchgears is important. Many works have been published since the past regarding simulation and modelling of defects in switchgears, PD measurement, fault diagnosis and ageing test on GIS based on the actual operating condition of the equipment. However, the models only replicated ideal conditions of the switchgears, which may not be similar to the actual site conditions. Hence, classification of faults in switchgears might not be effective by using modelling and simulation.

Several works have reported on fault diagnosis and condition monitoring of electrical equipment by using artificial intelligence. Support vector machine (SVM) was utilized for machine fault diagnosis in [11–16]. SVM is good at generalization that is able to yield good accuracy in condition monitoring classification. Artificial neural network (ANN) was also utilized for fault detection, classification and isolation in a transmission line system and prediction of failure analysis [17–19]. Other intelligent classifiers include condition-based reasoning, random forest fuzzy and expert system. However, the performance of the intelligent classifiers was not optimized, where some of the parameters used were based on the default values. Hence, there is still room for improvement in the performance of the classifiers.

The existing diagnosis techniques of abnormal location in switchgears have some drawbacks. In current practice, the abnormal location in switchgears is determined manually after measurements have been performed on-site. This could result in different diagnosis interpretation for the same measurement data by different personnel or expert. Also, manual interpretation is time consuming and might not be accurate. Thus, techniques that can determine the abnormal location within switchgears automatically, at higher accuracy and with less time-consuming are important. Although the application of intelligent classifiers has shown promising results, their performance can still be improved, especially if their parameters are tuned suitably to achieve the optimum performance.

In this work, classification of abnormal location in switchgears based on the on-site measurement data is proposed using hybrid gravitational search algorithm (GSA)-artificial intelligence (AI) techniques. The measurement data were obtained from ultrasound, transient earth voltage, temperature and sound sensors. The AI classifiers used include SVM and ANN. They are chosen because they have been commonly applied in past researches related to insulation diagnosis. ANN and SVM have several advantages, which make them a popular choice among other classifiers. Specifically, for ANN, it can store the information on the entire network, not on a database, able to work with incomplete knowledge, has fault tolerance, has a distributed memory, able to learn events and make decisions by commenting on similar events and has parallel processing capability. For SVM, it works well when there is a clear margin of separation between classes, effective in high dimensional spaces, effective when the number of dimensions is greater than the number of samples and is relatively memory efficient.

The performance of both classifiers is optimized by GSA. GSA is chosen in this work because it is easy to implement, it has fast convergence, low computational cost and the convergence rate can be controlled easily by the gravitational constant and the acceleration of the particles. Chaotic genetic algorithm (CGA), modified evolutionary programming (MEP) and the modified version of evolutionary particle swarm optimization (MEPSO) are also applied as the optimization techniques in this work for comparison purpose. These methods are the improved version of their original algorithm, which are particle swarm optimization (PSO), evolutionary programming (EP) and genetic algorithm (GA). They are chosen because they are the most widely used metaheuristic optimization techniques in literature. From the proposed method, the identification of abnormal location in switchgears can be performed automatically, quickly and with minimum error.

This paper is arranged as follows; the first section consists of introduction part including the past related works. The second section includes explanation on the project methodology, which includes artificial intelligence and optimization techniques. Section 3 reports the results obtained and their discussion and finally, the last section concludes the work outcomes.

## 2. Methodology

**Fig 1:**
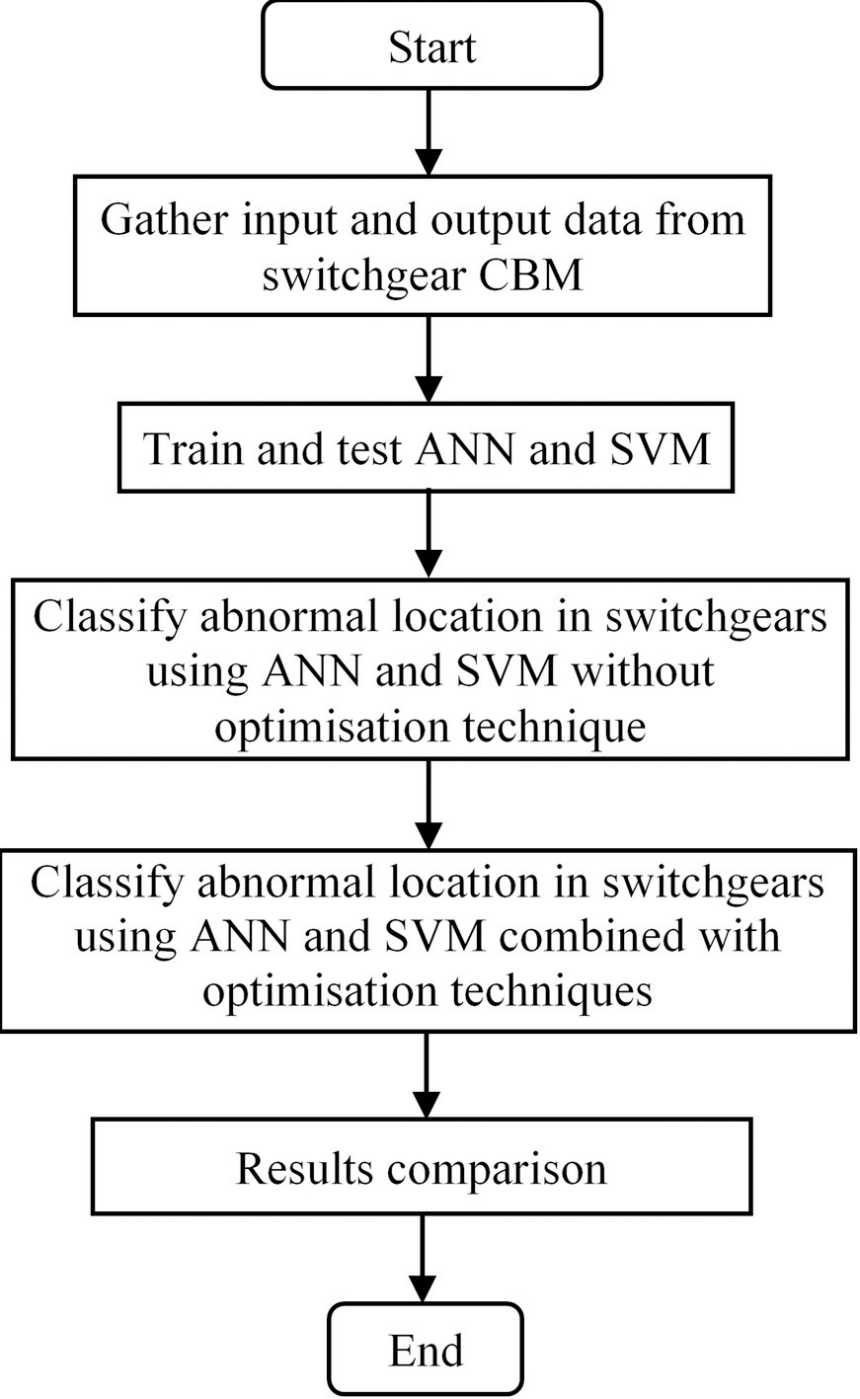
Flowchart of the overall methodology.

This section describes the methodology of the work, which is the classification of abnormal location in switchgears using hybrid gravitational search algorithm-artificial intelligence techniques. The overall flowchart of the work is shown in Fig 1. Firstly, the input and output data of condition based monitoring (CBM) from switchgears are gathered. Details regarding the input and output data are described in Section 2.1. Then, the intelligent classifiers, ANN and SVM are trained and tested using the input and output data. Details regarding the setting of the classifiers are described in Sections 2.2 and 2.3 for ANN and SVM respectively. At first, the abnormal locations in switchgears are classified using ANN and SVM without incorporating any optimization technique. After that, the abnormal locations in switchgears are classified again using ANN and SVM but combining with an optimization technique, which is gravitational search algorithm (GSA). Details regarding GSA algorithm and the setting for its parameters are described in Section 2.4. The objective function used for GSA is minimization of the classification percentage error of the abnormal location by AI classifiers. Finally, the results are presented and discussed and comparison between different techniques is performed. Other techniques used for comparison include chaotic genetic algorithm (CGA), modified evolutionary programming (MEP) and modified evolutionary particle swarm optimization (MEPSO).

### 2.1. Input and output data of switchgear condition monitoring

The CBM data of switchgears used as the input data for AI classifiers are ultrasound level, sound type (tracking, arcing, hissing and mechanical vibration), transient earth voltage (TEV) level and temperature. For every set of the measurement data, the output is taken as the abnormal location within the switchgears. The CBM data are gathered from an electrical utility from early 2017 to end of 2019. The abnormal locations include potential transformer compartment (bushing, insulation and connection), cable compartment (cable termination, lug and entry) and breaker compartment (spout, insulation and contact finger). Each of the sensors was installed at the potential transformer compartment, cable compartment and breaker compartment of the switchgears.

Table 1 summarizes the input and output data of switchgear abnormal locations. There is a total of 160 sets of input and output data. For every set of data, it consists of 4 types of input data, which are the CBM measurement data and 1 type of output data, which is the abnormal location and normal condition. There are 40 sets of data for every output type. For classification, from the overall data, 70% of the data are used for training while the rest are used for testing. *k*-fold cross validation is also used for classification, where *k* is chosen to be 4 according to the number of data sets available.

**Table 1:** CBM data from switchgears for classification of abnormal location.

Input data		Output data
Measurement data	Range or data type	
Ultrasound level	1–30 dB	Potential transformer (PT) compartment
Sound Type	Mechanical vibration	
	Tracking sound	Circuit breaker (CB) compartment
		Cable compartment
	Arcing sound	
	Hissing sound	
		Normal condition
TEV level	1–20 dB	
Temperature	30–46°C	

### 2.2. Artificial Neural Network (ANN)

ANN loosely models the neuron functions in a human’s brain for pattern recognition [20–24]. It can interpret data via clustering, machine perception or labeling input data. Its ability to learn, parallel processing and generalization have made ANN suitable for various systems. The application of ANN for classifying patterns is very common. It acts as a classification layer on top of the data stored. Data without label are grouped based on similarity between the input data. The data are then classified after the dataset are labelled to train [25, 26].

ANN is able to perform non-linear statistical modeling and can be an alternative to logistic regression [27]. The benefits of ANN are it does not require many statistical training and it is able to determine possible interactions between variables of predictor. It is able to determine non-linear complex linkage between dependent and independent variables. It is also allowed to have many algorithms for training. The disadvantages of ANN are higher computational burden, tend to over-fitting and the model development in empirical nature.

In this work, back-propagation network (BPN) is applied for training. BPN has a generalized feed-forward multi-layer network delta protocol. BPN passes two steps, updating the weight and propagation. The interconnected BPN layers are the input, hidden and output layers. In BPN, the input is propagated forward through each of the layers at various weights before reaching the output layer. At the training stage, the output is compared with the expected output. The error is fed again into the network for weight adjustment until the error between the expected output and determined output is very small.

**Fig 2:**
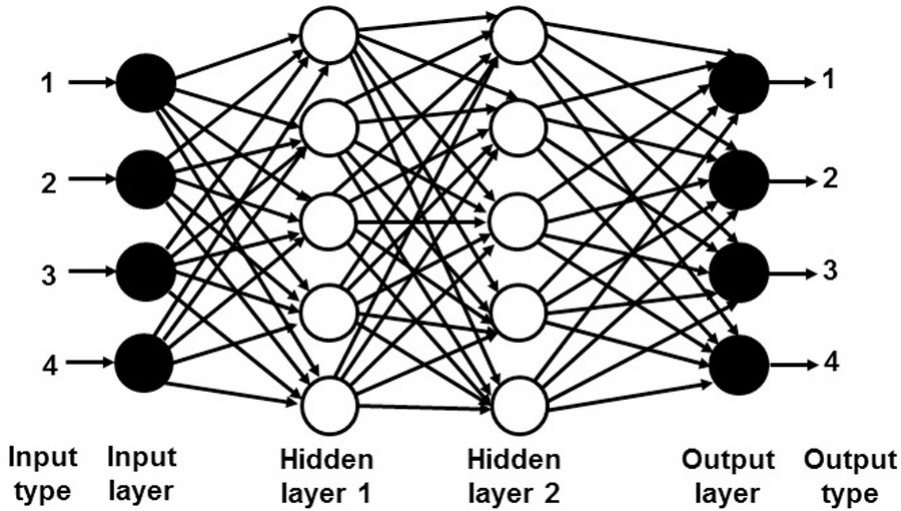
ANN structure used in this work.

In the hidden layer, sigmoid transfer function is applied due to the non-linearity and it can be used with feed-forward backpropagation-learning algorithm. Gradient Descent learning function and Lavenberg-Marquart training function with bias learning function and momentum weight were applied due to their simplicity, robustness and speed. After several trials, it was found that two hidden layers and five neurons in each hidden layer yield the highest accuracy of the output. Increasing the number of neurons and hidden layers beyond this does not improve the results further. Although one hidden layer is adequate for mapping of non-linear data, 2 hidden layers of ANN are better in terms of the accuracy, iteration and complexity. The ANN structure with more than two hidden layers can overcome slow convergence but it does not improve the accuracy of the results further. Fig 2 shows the ANN structure used in this work, which consists of 4 types of input data, 2 hidden layers and 4 types of output data. Every hidden layer consists of 5 neurons. For the proposed hybrid GSA-ANN, the ANN performance is optimized by setting the momentum constant (MC) and learning rate (LR) as the variables in the optimization algorithm.

### 2.3. Support Vector Machine (SVM)

**Fig 3:**
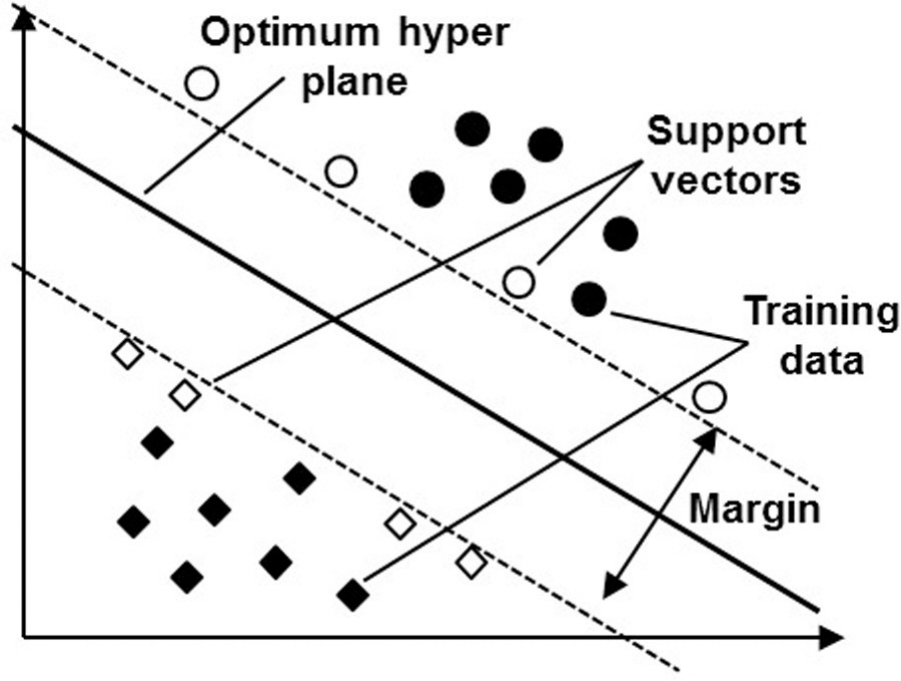
Concept of classification by SVM (unfilled elements represent support vectors while filled elements represent training data).

SVM is a technique according to the theory of statistical learning applied to determine the decisive boundary via separating different classes and increasing the margin [28–30]. SVM is fit for non-linear data set problems and less number of training data but with huge number of input. Fig 3 conceptualizes SVM, where a hyperplane is created to maximize the support vectors, which are also called as margin data points [31, 32].

In this work, Gaussian radial basis function (RBF) kernel is chosen because the problem to be solved is more than one class. Gaussian RBF is able to project the features of input into an infinite feature space, enabling the training data to be linearly separated. The equation related to RBF is

$$F\left(x,x'\right)=\exp\left(\frac{{\left\|x-x'\right\|}^{2}}{2\sigma^{2}}\right)$$ (1)

where Gaussian RBF kernel scaling factor is denoted by *σ* and support vectors are denoted by *x’* and *x*. For the proposed hybrid GSA-SVM in this work, the SVM performance is optimized by taking the box constraint *c* and *σ* as the variables in the optimization algorithm. These parameters can control the performance of SVM. Accordingly, a huge value of *c* yields a smaller margin of hyperplane but it is able to classify all training points exactly. This yields stricter classification and also overfitting. If *c* is small, a larger margin of hyperplane is developed, yielding underfitting.

### 2.4. Gravitational Search Algorithm (GSA)

**Fig 4:**
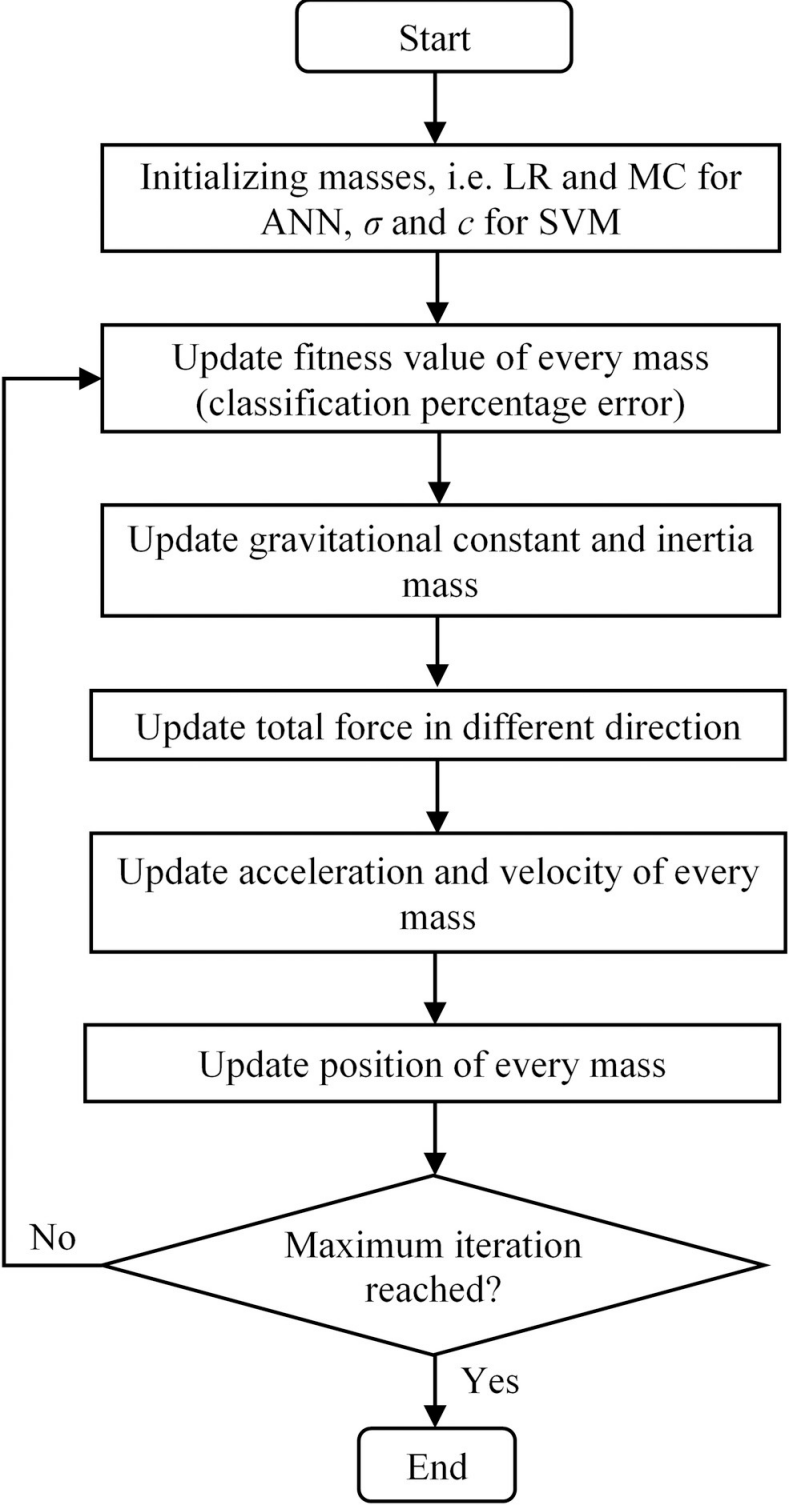
Flowchart of the proposed GSA-AI technique.

GSA applies motion law and Newton’s law of gravity concepts. Each of the particles is attracted to each other via a gravity force [33]. Masses are the candidate solutions for the population that possess their own masses of inertial, gravitational, passive, active and position. The global movement happens due to the gravity force that is attracting each of the masses to each other. Smaller masses will be propagating towards larger and slower masses, which represent a better solution compared to the latest solution. Fig 4 depicts the overall flowchart of the proposed GSA-AI technique in the methodology. The explanation of the flowchart is as follows:

#### Initialization

Random initial masses of the population are created, which are momentum constant (MC) and learning rate (LR) for ANN and the scaling factor *σ* and box constraint *c* for SVM. For every set of LR and MC for ANN and *σ* and *c* for SVM, the fitness function value is calculated, which is minimization of the classification percentage error of the abnormal location by AI classifiers.

#### Updating constant

The gravitational constant *G* at iteration *i* is calculated using

$$G\left(i\right)=G_{0}\;\text{exp}\left(-\alpha \;i\;/\;i_{max}\right)$$ (2)

where *α* is a constant, *G*_0_ is the initial constant of gravitational, *i* is the iteration and *i*_*max*_ is the maximum iteration.

#### Updating mass

The inertia mass *M*_*j*_ is updated by

$$M_{j}\left(i\right)=m_{j}\left(i\right)\;/\;\left[\Sigma^{N}{{}_{i}}_{=1}\;m_{j}\left(i\right)\right]$$ (3)

where *m*_*j*_ is the mass *j* calculated by

$$m_{j}\left(i\right)=\left[fit_{j}\left(i\right)-worst\left(i\right)]\;/[best\left(i\right)-worst\left(i\right)\right]$$ (4)

where *fit*_*j*_ is the fitness of mass *j*. The *worst*(*i*) is found by max(*fit*_*j*_(*i*)) while *best*(*i*) is found by min(*fit*_*j*_(*i*)).

#### Updating total force

The gravitational force *F*_*jk*_ acting on mass *j* by mass *k* is

$$F_{jk}\left(i\right)=G\left(i\right)M_{j}\left(i\right)\left[M_{k}\left(i\right)-M_{j}\left(i\right)\right]/\left[R_{jk}{\left(i\right)}^{\text{Rpow}}+\varepsilon \right]$$ (5)

where *R*_*jk*_ is the Euclidian distance between masses *j* and *k*, *M*_*j*_ is the inertia mass *j*, *M*_*k*_ is the inertia mass *k*, *R*_*pow*_ is the Euclidian distance power and *ε* is a constant.

#### Updating mass velocity

The acceleration *a*_*j*_ of mass *j* is updated using

$$a_{j}\left(i\right)=F_{j}\left(i\right)\;/\;M_{j}\left(i\right)$$ (6)

where *F*_*j*_ is the total force acting on mass *j* determined by

$$F_{j}\left(i\right)=\Sigma^{N}{{}_{k}}_{\neq j}\;rand\left(0,1\right)\;F_{jk}\left(i\right)$$ (7)

where *rand*(0,1) is a random number between [0, 1]. The new velocity of the mass is calculated using

$$v_{j}\left(i+1\right)=rand\;v_{j}\left(i\right)+a_{j}\left(i\right)$$ (8)

#### Updating mass position

The new position of every mass *j* is determined by

$$p_{j}\left(i+1\right)=p_{j}\left(i\right)+v_{j}\left(i+1\right)$$ (9)

## 3. Results and discussion

In this section, the results obtained from the classification of abnormal location in switchgears using ANN alone, SVM alone, GSA-ANN and GSA-SVM are reported and discussed. For comparison purposes, the results using PSO, EP and GA combined with ANN and SVM respectively are also reported.

### 3.1. Classification using ANN and SVM alone

Tables 2 to 5 show the classification accuracy results using ANN and SVM alone, where the performance is not optimized by the optimization technique, GSA. The classification accuracy is calculated in terms of correct classification of abnormal location in switchgears against the actual location. The overall classification accuracy is the average accuracy calculated from each of the test data.

**Table 2:** Classification results using ANN based on 70:30 ratio for training and testing data.

Output Test data	Number of output classified				Classification accuracy (%)
	PT	CB	Cable	Normal	
PT	9	0	1	0	90
CB	2	8	0	0	80
Cable	0	0	10	0	100
Normal	0	2	0	8	80
Overall classification accuracy (%)					87.50

**Table 3:** Classification results using SVM based on 70:30 ratio for training and testing.

Output Test data	Number of output classified				Classification accuracy (%)
	PT	CB	Cable	Normal	
PT	9	0	3	0	75
CB	0	11	0	1	91.67
Cable	0	0	11	1	91.67
Normal	0	0	0	12	100
Overall classification accuracy (%)					89.58

**Table 4:** Classification results using ANN based on 4-fold cross validation.

Fold	Output Test data	Number of output classified				Classification accuracy (%)
		PT	CB	Cable	Normal	
1	PT	8	1	1	0	80
	CB	0	10	0	0	100
	Cable	2	0	8	0	80
	Normal	0	0	0	10	100
2	PT	6	2	2	0	60
	CB	0	7	3	0	70
	Cable	1	0	9	0	90
	Normal	0	0	0	10	100
3	PT	7	3	0	0	70
	CB	0	10	0	0	100
	Cable	0	1	9	0	90
	Normal	0	0	1	9	90
4	PT	8	2	0	0	80
	CB	3	7	0	0	70
	Cable	0	0	10	0	100
	Normal	0	0	2	8	80
Overall classification accuracy (%)						85.00

**Table 5:** Classification results using SVM based on 4-fold cross validation.

Fold	Output Test data	Number of output classified				Classification accuracy (%)
		PT	CB	Cable	Normal	
1	PT	6	0	0	4	60
	CB	0	10	0	0	100
	Cable	3	0	6	1	60
	Normal	0	0	0	10	100
2	PT	8	0	1	1	80
	CB	0	7	0	3	70
	Cable	1	0	9	0	90
	Normal	0	0	0	10	100
3	PT	10	0	0	0	100
	CB	0	8	0	2	80
	Cable	0	0	9	1	90
	Normal	0	0	0	10	100
4	PT	7	0	3	0	70
	CB	0	9	0	1	90
	Cable	0	0	10	0	100
	Normal	0	0	0	10	100
Overall classification accuracy (%)						86.88

Tables 2 and 3 show the number of output classified according to the test data and classification accuracy using ANN and SVM based on 70% of the data sets for training and the remaining data for testing. The overall classification accuracy using ANN is 87.50% while for SVM is 89.58%. For ANN, the classification accuracies according to the test data are 90% (PT compartment), 83% (CB compartment), 100% (cable compartment) and 80% (normal). For SVM, the classification accuracies according to the test data are 75% (PT compartment), 91.67% (CB compartment), 91.67% (cable compartment) and 100% (normal).

Tables 4 and 5 show the number of output classified according to the test data and classification accuracy using ANN and SVM based on 4-fold cross validation. The overall classification accuracy using ANN is 85% while for SVM is 86.88%. These values are near to the accuracy using 70:30 ratio for training and testing data, which are 87.5% and 89.58% for ANN and SVM respectively. For unoptimized classifiers, SVM yields slightly higher accuracy than ANN, which is higher by around 2%.

### 3.2. Classification using GSA-ANN and GSA-SVM

Tables 6–9 show the numbers of output classified according to the test data and classification accuracy using the proposed hybrid optimization algorithm-intelligent classifiers, which are GSA-ANN and GSA-SVM based on 70:30 ratio for training and testing data and 4-fold cross validation. For ANN, the classification accuracies using GSA-ANN are 97.92% and 99.38% based on 70:30 ratio for training and testing data and 4-fold cross validation respectively. Compared to the ANN technique alone, GSA-ANN shows an improvement between 10% and 14% in the classification accuracy. Thus, by incorporating GSA with ANN, the ANN performance is improved by varying its learning rate and momentum constant. For SVM, the classification accuracies using GSA-SVM are 95.83% and 96.88% based on 70:30 ratio for training and testing data and 4-fold cross validation respectively. Compared to the SVM technique alone, GSA-SVM shows an improvement between 6% and 10% in the classification accuracy. Thus, by incorporating GSA with SVM, the performance of SVM is also improved by varying its box constraint and Gaussian RBF kernel scaling factor.

**Table 6:** Classification results using GSA-ANN based on 70:30 ratio for training and testing data.

Output Test data	Number of output classified				Classification accuracy (%)
	PT	CB	Cable	Normal	
PT	11	0	1	0	91.67
CB	0	12	0	0	100
Cable	0	0	12	0	100
Normal	0	0	0	12	100
Overall classification accuracy (%)					97.92

**Table 7:** Classification results using GSA-SVM based on 70:30 ratio for training and testing data.

Output Test data	Number of output classified				Classification accuracy (%)
	PT	CB	Cable	Normal	
PT	12	0	0	0	100
CB	2	10	0	0	83.33
Cable	0	0	12	0	100
Normal	0	0	0	12	100
Overall classification accuracy (%)					95.83

**Table 8:** Classification results using GSA-ANN based on 4-fold cross validation.

Fold	Output Test data	Number of output classified				Classification accuracy (%)
		PT	CB	Cable	Normal	
1	PT	10	0	0	0	100
	CB	0	10	0	0	100
	Cable	1	0	9	0	90
	Normal	0	0	0	10	100
2	PT	10	0	0	0	100
	CB	0	10	0	0	100
	Cable	0	0	10	0	100
	Normal	0	0	0	10	100
3	PT	10	0	0	0	100
	CB	0	10	0	0	100
	Cable	0	0	10	0	100
	Normal	0	0	0	10	100
4	PT	10	0	0	0	100
	CB	0	10	0	0	100
	Cable	0	0	10	0	100
	Normal	0	0	0	10	100
Overall classification accuracy (%)						99.38

**Table 9:** Classification results using GSA-SVM based on 4-fold cross validation.

Fold	Output Test data	Number of output classified				Classification accuracy (%)
		PT	CB	Cable	Normal	
1	PT	9	0	0	1	90
	CB	0	10	0	0	100
	Cable	2	0	8	0	80
	Normal	0	0	0	10	100
2	PT	10	0	0	0	100
	CB	0	10	0	0	100
	Cable	1	0	9	0	90
	Normal	0	0	0	10	100
3	PT	10	0	0	0	100
	CB	0	10	0	0	100
	Cable	1	0	9	0	90
	Normal	0	0	0	10	100
4	PT	10	0	0	0	100
	CB	0	10	0	0	100
	Cable	0	0	10	0	100
	Normal	0	0	0	10	100
Overall classification accuracy (%)						96.88

From the comparison of ANN and SVM between with and without optimization, the accuracy result of ANN is better than SVM after optimization with GSA. Also, the improvement brought by GSA on the performance of ANN is more significant compared to SVM. This is due to SVM works by placing data points above and below the classifying hyperplane. Hence, variation of its parameters, i.e. *c* and *σ*, is not affecting its performance so much. However, for ANN, its performance is strongly dependent on the momentum constant (MC) and learning rate (LR). LR controls the amount of the weight update during training while MC controls the convergence rate of ANN. Thus, variation of these parameters is affecting the performance of ANN significantly.

**Fig 5:**
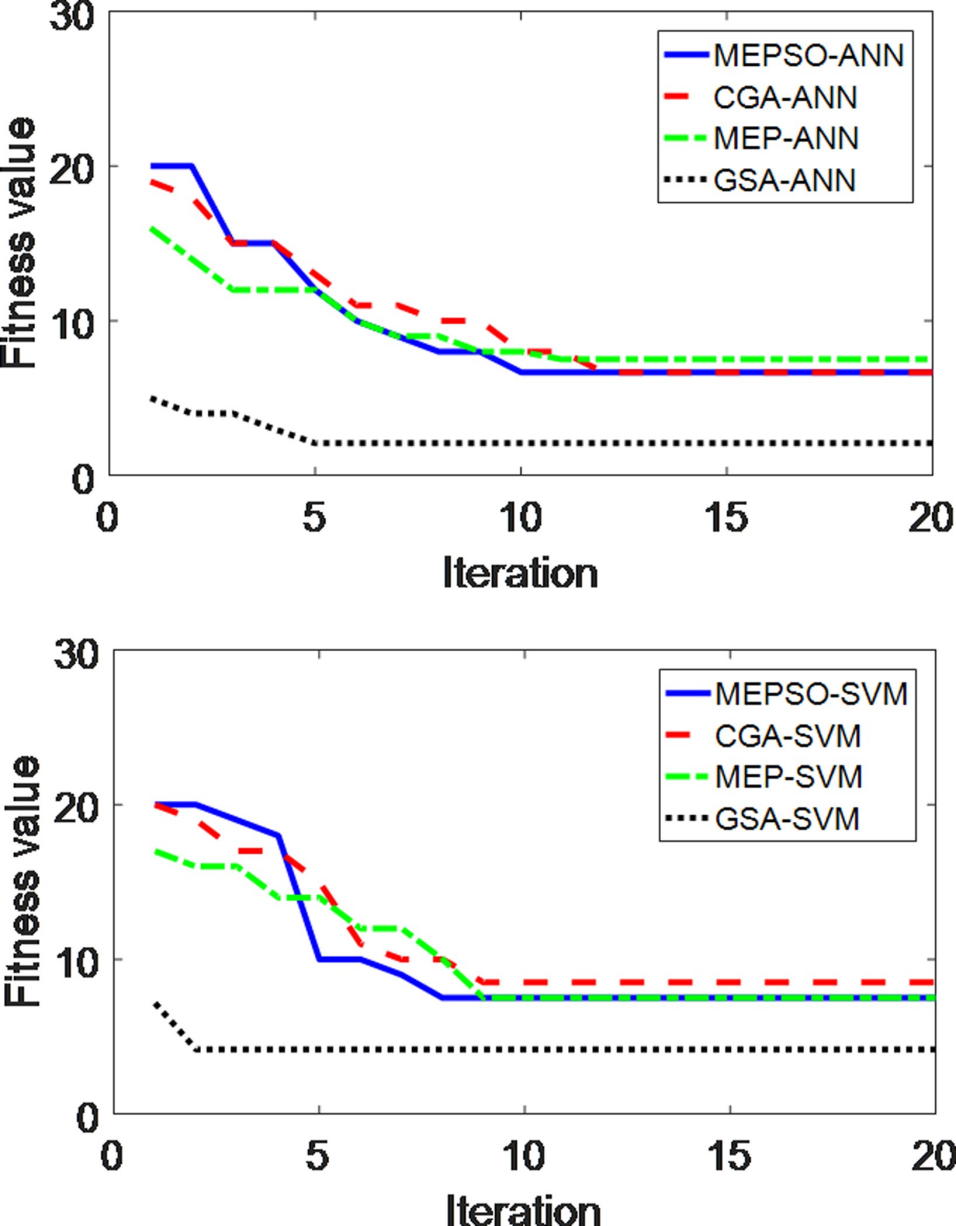
Convergence curve of different algorithms combined with (a) ANN and (b) SVM using 70:30 ratio for training and testing data.

Table 10 shows the comparison of the classification accuracy results between hybrid GSA-AI and several well-known metaheuristic algorithms. They include chaotic genetic algorithm (CGA) [34, 35], modified evolutionary programming (MEP) [36] and modified evolutionary particle swarm optimization (MEPSO) [37]. From Table 10, the proposed GSA-AI yields higher accuracy than the other algorithms. This is due to GSA is able to search near optimum global solution, making it different from other nature inspired algorithms. GSA-SVM converges faster than GSA-ANN and the other algorithms used for comparison as shown in Fig 5. Referring to the total execution time in Table 10, the time for GSA-ANN and GSA-SVM is faster than the other algorithms used for comparison. Thus, GSA can be considered better than the other three metaheuristic algorithms for classification of abnormal location in switchgears.

**Table 10:** Results comparison between different algorithms.

Algorithm	70:30 ratio for training and testing data			4-fold cross validation
	Classification accuracy (%)	Iteration of convergence	Total execution time (sec)	Classification accuracy (%)
ANN	87.50	N/A	N/A	85.00
SVM	89.58	N/A	N/A	86.88
MEPSO-ANN	93.33	10	204.9544	94.38
MEPSO-SVM	92.50	8	35.5439	93.13
CGA-ANN	93.33	12	101.8458	90.63
CGA-SVM	91.50	9	24.1253	91.25
MEP-ANN	92.50	11	101.3225	91.88
MEP-SVM	92.50	9	25.6904	91.88
GSA-ANN	97.92	5	97.0383	99.38
GSA-SVM	95.83	2	20.0036	96.88

Table 11 shows the statistical test of non-parametric according to McNemar’s test for the proposed method. Using similar data sets of input and output, the *p*-values calculated between the proposed method and the other methods are less than the significance threshold *α*, which is set to 0.05. Since *p*-values are less than *α*, this indicates that a significant difference exists in the results found between the proposed method and the other methods.

**Table 11:** Statistical test of non-parametric using McNemar’s test for the method proposed.

Comparison between		*p*-value
Method 1	Method 2	
GSA-ANN	ANN	0.0144
GSA-ANN	MEPSO-ANN	0.0106
GSA-ANN	CGA-ANN	0.0376
GSA-ANN	MEP-ANN	0.0077
GSA-SVM	SVM	0.0309
GSA-SVM	MEPSO-SVM	0.0186
GSA-SVM	CGA-SVM	0.0500
GSA-SVM	MEP-SVM	0.0352

## 4. Conclusions

Classification of abnormal location in switchgears has been successfully proposed using hybrid gravitational search algorithm (GSA)-artificial intelligence (AI) techniques. The AI classifiers used include support vector machine (SVM) and artificial neural network (ANN). It was found that GSA has successfully improved the performance of both classifiers by tuning their parameters to suitable values. Without optimization by GSA, SVM yields slightly higher accuracy than ANN, which is more than 2%. However, after performance optimization by GSA, ANN yields slightly higher accuracy than SVM, which is in the range of 97%-99% compared to 95%-97 for SVM. On the other hand, GSA-SVM converges faster than GSA-ANN. Comparison between GSA-AI, chaotic genetic algorithm (CGA)-AI, modified evolutionary programming (MEP)-AI and modified evolutionary particle swarm optimization (MEPSO)-AI shows that GSA-AI achieves higher accuracy and has faster convergence than the other optimization techniques. A non-parametric statistical test based on McNemar’s test shows a significant difference exists in the results found between the proposed method and the other methods. Therefore, the proposed hybrid GSA-AI methods can be recommended in the actual practice to determine the abnormal location in switchgears automatically, quickly and with lower error. In future work, the effect of the combination methods, such as intelligent classifiers with regression and parallel structure approach with different voting rules, intelligent classifiers other than ANN and SVM and optimization algorithms other than GSA can also be explored for classification of abnormal location in switchgears.

## Supporting information

S1 Data (PDF)

## References

[1] Bakar A. H. A., Illias H. A., Othman M. K., and Mokhlis H., “Identification of failure root causes using condition based monitoring data on a 33kV switchgear,” International Journal of Electrical Power & Energy Systems, vol. 47, pp. 305–312, 2013.

[2] Behrmann G., Koltunowicz W., and Schichler U., “State of the Art in GIS PD Diagnostics,” in 2018 Condition Monitoring and Diagnosis (CMD), 2018, pp. 1–6.

[3] Zhang C., Dong M., Ren M., Huang W., Zhou J., Gao X., et al., “Partial Discharge Monitoring on Metal-Enclosed Switchgear with Distributed Non-Contact Sensors,” Sensors, vol. 18, p. 551, 2018. doi: 10.3390/s18020551 29439475 PMC5855104

[4] Wang B. and Lin T.-N., “A Passive RFID Sense Tag for Switchgear Thermal Monitoring in Power Grid,” Qatar Foundation Annual Research Conference Proceedings, vol. 2018, p. ICTPD712, 2018.

[5] Budyn M., Karandikar H., and G Urmson M., “Switchgear Condition Monitoring,” in CIGRÉ Conference on Power Systems, Vancouver, Canada, 2010.

[6] Li J., Han X., Liu Z., and Yao X., “A Novel GIS Partial Discharge Detection Sensor With Integrated Optical and UHF Methods,” IEEE Transactions on Power Delivery, vol. 33, pp. 2047–2049, 2018.

[7] X. Zeng, H. Li, Y. Lu, and Y. Chen, "Online monitoring of partial discharge in high voltage switchgear using a differential electric field sensor," in 2017 IEEE Conference on Electrical Insulation and Dielectric Phenomenon (CEIDP), 2017, pp. 385–388.

[8] Straumann U. and Stoller P. C., “The impact of partly liquefied SF6 on the dielectric performance of SF6-insulated switchgear,” IEEE Transactions on Dielectrics and Electrical Insulation, vol. 26, pp. 137–145, 2019.

[9] T. Juliandhy, T. Haryono, Suharyanto, and I. Perdana, "Comparison of CF3CHCl2 gas with SF6gas as an alternative substitute for gas insulated switchgear equipment," in 2017 International Conference on High Voltage Engineering and Power Systems (ICHVEPS), 2017, pp. 198–203.

[10] Sahoo A., Subramaniam A., Bhandari S., and Panda S. K., “A review on condition monitoring of GIS,” in 2017 International Symposium on Electrical Insulating Materials (ISEIM), 2017, pp. 543–546.

[11] Krishna K. R. and Ramachandran K. I., “Machinery Bearing Fault Diagnosis Using Variational Mode Decomposition and Support Vector Machine as a Classifier,” IOP Conference Series: Materials Science and Engineering, vol. 310, p. 012076, 2018.

[12] Benkedjouh T., Chettibi T., Saadouni Y., and Afroun M., “Gearbox Fault Diagnosis Based on Mel-Frequency Cepstral Coefficients and Support Vector Machine,” in Computational Intelligence and Its Applications, Cham, 2018, pp. 220–231.

[13] Zheng J., Pan H., and Cheng J., “Rolling bearing fault detection and diagnosis based on composite multiscale fuzzy entropy and ensemble support vector machines,” Mechanical Systems and Signal Processing, vol. 85, pp. 746–759, 2017.

[14] Chothani N. G., Bhalja B. R., and Parikh U. B., “New fault zone identification scheme for busbar using support vector machine,” IET Generation, Transmission and Distribution, vol. 5, pp. 1073–1079, 2011.

[15] Aziz O., Klenk J., Schwickert L., Chiari L., Becker C., Park E. J., et al., “Validation of accuracy of SVM-based fall detection system using real-world fall and non-fall datasets,” PLOS ONE, vol. 12, p. e0180318, 2017. doi: 10.1371/journal.pone.0180318 28678808 PMC5498034

[16] Eskandarpour R. and Khodaei A., “Leveraging Accuracy-Uncertainty Tradeoff in SVM to Achieve Highly Accurate Outage Predictions,” IEEE Transactions on Power Systems, vol. 33, pp. 1139–1141, 2018.

[17] Zhao L., Goh S. H., Chan Y. H., Yeoh B. L., Hu H., Thor M. H., et al., “Optimization of an Artificial Neural Network System for the Prediction of Failure Analysis Success,” Microelectronics Reliability, vol. 92, pp. 136–142, 2019.

[18] Goh S. H., “Yield-oriented logic failure characterization for FA prioritization,” in EDFA Magazine. vol. 16, ed: ASM, 2014, pp. 4–12.

[19] Afram A., Janabi-Sharifi F., Fung A. S., and Raahemifar K., “Artificial neural network (ANN) based model predictive control (MPC) and optimization of HVAC systems: A state of the art review and case study of a residential HVAC system,” Energy and Buildings, vol. 141, pp. 96–113, 2017.

[20] Lek S. and Park Y. S., "Artificial Neural Networks,” in Encyclopedia of Ecology, Jørgensen S. E. and Fath B. D., Eds., ed Oxford: Academic Press, 2008, pp. 237–245.

[21] Illias H. A., Chai X. R., Abu Bakar A. H., and Mokhlis H., “Transformer Incipient Fault Prediction Using Combined Artificial Neural Network and Various Particle Swarm Optimisation Techniques,” PLOS ONE, vol. 10, p. e0129363, 2015. doi: 10.1371/journal.pone.0129363 26103634 PMC4478012

[22] Fan X., Sun T., Chen W., and Fan Q., “Deep neural network based environment sound classification and its implementation on hearing aid app,” Measurement, vol. 159, p. 107790, 2020.

[23] Li Y., Li J., Huang J., and Zhou H., “Fitting analysis and research of measured data of SAW micro-pressure sensor based on BP neural network,” Measurement, vol. 155, p. 107533, 2020. 10.1016/j.measurement.2020.107533 PMC8580376 34764527

[24] Zaji A. H., Bonakdari H., Khameneh H. Z., and Khodashenas S. R., “Application of optimized Artificial and Radial Basis neural networks by using modified Genetic Algorithm on discharge coefficient prediction of modified labyrinth side weir with two and four cycles,” Measurement, vol. 152, p. 107291, 2020.

[25] Ajjolli Nagaraja A., Fontaine N., Delsaut M., Charton P., Damour C., Offmann B., et al., “Flux prediction using artificial neural network (ANN) for the upper part of glycolysis,” PLOS ONE, vol. 14, p. e0216178, 2019. doi: 10.1371/journal.pone.0216178 31067238 PMC6505829

[26] Walczak S. and Velanovich V., “Prediction of perioperative transfusions using an artificial neural network,” PLOS ONE, vol. 15, p. e0229450, 2020. doi: 10.1371/journal.pone.0229450 32092108 PMC7039514

[27] Marouf A. and Abu-Naser S., “Predicting Antibiotic Susceptibility Using Artificial Neural Network,” International Journal for Academic Development, vol. 2, pp. 1–5, 2018.

[28] Xiao R., Hu Q., and Li J., “Leak detection of gas pipelines using acoustic signals based on wavelet transform and Support Vector Machine,” Measurement, vol. 146, pp. 479–489, 2019.

[29] Wang Z. and Zhu D., “An accurate detection method for surface defects of complex components based on support vector machine and spreading algorithm,” Measurement, vol. 147, p. 106886, 2019.

[30] Abdalmalak K. A. and Gallardo-Antolín A., “Enhancement of a text-independent speaker verification system by using feature combination and parallel structure classifiers,” Neural Computing & Applications, vol. 29, pp. 637–651, 2018.

[31] Yamamoto M., Bagarinao E., Kushima I., Takahashi T., Sasabayashi D., Inada T., et al., “Support vector machine-based classification of schizophrenia patients and healthy controls using structural magnetic resonance imaging from two independent sites,” PLOS ONE, vol. 15, p. e0239615, 2020. doi: 10.1371/journal.pone.0239615 33232334 PMC7685428

[32] Chu M., Liu X., Gong R., and Zhao J., “Support vector machine with quantile hyper-spheres for pattern classification,” PLOS ONE, vol. 14, p. e0212361, 2019. doi: 10.1371/journal.pone.0212361 30768635 PMC6377146

[33] Rashedi E., Nezamabadi-pour H., and Saryazdi S., “GSA: A Gravitational Search Algorithm,” Information Sciences, vol. 179, pp. 2232–2248, 2009.

[34] Chiang H.-D., Chen L.-G., Liu R.-P., and Dong N., “Group-based chaos genetic algorithm and non-linear ensemble of neural networks for short-term load forecasting,” IET Generation, Transmission & Distribution, vol. 10, pp. 1440–1447, 2016.

[35] Fuertes G., Vargas M., Alfaro M., Soto-Garrido R., Sabattin J., and Peralta M. A., “Chaotic genetic algorithm and the effects of entropy in performance optimization,” Chaos: An Interdisciplinary Journal of Nonlinear Science, vol. 29, p. 013132, 2019. doi: 10.1063/1.5048299 30709130

[36] Jena C., Basu M., and Panigrahi C. K., “Modified evolutionary programming for short-term hydrothermal scheduling,” International Journal of Power and Energy Conversion, vol. 9, pp. 384–408, 2018.

[37] Illias H. A., Chai X. R., and Abu Bakar A. H., “Hybrid modified evolutionary particle swarm optimisation-time varying acceleration coefficient-artificial neural network for power transformer fault diagnosis,” Measurement, vol. 90, pp. 94–102, 2016.

