# Supplementary material for: Classification of abnormal location in medium voltage switchgears using hybrid gravitational search algorithm-artificial intelligence
Source: PLoS One. 2021 Jul 1;16(7):e0253967. doi: 10.1371/journal.pone.0253967 (PMC8248718; doi:10.1371/journal.pone.0253967)
Supplement: S1 Data — (PDF) [file pone.0253967.s001.pdf]

Ultrasound	Sound type	TEV level	Temperature	Output
-1	4	5	33	1
5	4	5	44	1
6	1	7	35	1
7	4	5	33	1
10	1	7	33	1
10	1	7	33	1
10	4	5	34	1
10	4	5	35	1
10	1	7	36	1
11	4	5	39	1
13	4	5	46	1
17	2	5	36	1
20	2	7	39	1
20	2	7	41	1
6	2	5	33	1
10	2	6	33	1
10	2	5	34	1
10	2	7	35	1
20	2	8	33	1
20	1	7	34	1
3	4	5	33	1
4	4	5	44	1
7	2	7	35	1
8	4	5	33	1
11	2	7	33	1
11	2	7	33	1
11	4	5	34	1
11	4	5	35	1
11	2	7	36	1
12	4	5	39	1
14	4	5	46	1
18	4	5	36	1
21	2	7	39	1
21	2	7	41	1
7	2	6	33	1
11	2	5	33	1
11	2	6	34	1
11	2	7	35	1
21	2	6	33	1
21	2	6	34	1
1	1	10	31	2
1	1	11	33	2
2	1	10	34	2
2	1	12	35	2
2	4	11	36	2
2	1	12	41	2
3	1	10	34	2
2	1	10	46	2
3	4	10	33	2
3	1	11	36	2
2	4	12	41	2
2	1	13	44	2
3	1	13	46	2
2	4	13	31	2
2	4	11	37	2
3	1	12	44	2
2	1	11	37	2
1	3	10	38	2

1	4	10	44	2
3	4	11	33	2
2	4	12	35	2
3	4	13	38	2
2	4	14	44	2
2	3	10	46	2
1	1	10	31	2
2	1	11	33	2
1	1	10	34	2
2	1	11	35	2
2	1	11	36	2
3	1	11	41	2
5	1	12	31	2
6	1	12	33	2
7	1	11	34	2
7	1	10	35	2
9	1	13	36	2
4	1	13	41	2
3	1	12	31	2
4	1	12	33	2
3	1	11	34	2
2	1	11	35	2
20	1	4	31	3
19	2	5	33	3
20	1	4	33	3
19	3	5	34	3
19	1	4	34	3
18	1	4	34	3
18	1	4	35	3
19	2	4	36	3
20	2	4	37	3
20	1	4	34	3
21	1	4	35	3
22	1	4	36	3
20	1	4	37	3
19	1	4	31	3
19	4	4	33	3
19	1	4	35	3
20	1	4	36	3
21	1	4	37	3
22	1	4	38	3
19	3	3	39	3
18	3	4	41	3
18	1	4	43	3
19	3	3	47	3
18	1	4	31	3
19	1	4	34	3
20	3	2	37	3
21	3	1	38	3
20	4	1	39	3
19	1	4	43	3
18	1	4	47	3
19	1	4	31	3
18	1	4	33	3
18	1	4	34	3
18	1	4	35	3
18	3	2	36	3
18	4	3	38	3
18	3	2	39	3

19	1	4	41	3
19	1	4	43	3
19	1	4	46	3
3	1	1	31	4
2	1	2	30	4
2	1	1	31	4
1	1	1	30	4
1	1	2	30	4
2	1	2	31	4
2	1	2	32	4
3	1	1	31	4
2	1	2	31	4
2	1	1	32	4
1	1	1	31	4
1	1	2	31	4
2	1	2	32	4
2	3	2	30	4
2	1	1	30	4
2	1	2	30	4
3	1	2	31	4
3	1	2	32	4
4	1	1	31	4
3	1	2	31	4
3	1	1	32	4
2	1	1	31	4
2	1	2	31	4
3	1	2	32	4
3	3	2	30	4
3	2	1	31	4
2	2	2	30	4
2	2	1	31	4
1	2	1	30	4
1	2	2	30	4
2	2	2	31	4
2	2	2	32	4
3	2	1	31	4
2	2	2	31	4
2	2	1	32	4
1	2	1	31	4
3	3	1	31	4
3	3	2	31	4
2	1	3	31	4
2	2	3	31	4
